# The Effects of Granulocyte Colony-Stimulating Factor in Patients with a Large Anterior Wall Acute Myocardial Infarction to Prevent Left Ventricular Remodeling: A 10-Year Follow-Up of the RIGENERA Study

**DOI:** 10.3390/jcm9041214

**Published:** 2020-04-23

**Authors:** Antonio Maria Leone, Domenico D’Amario, Francesco Cannata, Francesca Graziani, Josip A. Borovac, Giuseppe Leone, Valerio De Stefano, Eloisa Basile, Andrea Siracusano, Leonarda Galiuto, Gabriella Locorotondo, Italo Porto, Rocco Vergallo, Francesco Canonico, Attilio Restivo, Antonio Giuseppe Rebuzzi, Filippo Crea

**Affiliations:** 1 Department of Cardiovascular Sciences, Fondazione Policlinico Universitario Agostino Gemelli, IRCCS, Università Cattolica del Sacro Cuore, Largo A. Gemelli 8, 00168 Rome, Italy; domenico.damario@gmail.com (D.D.); francesca.graziani4@gmail.com (F.G.); gabryloc@hotmail.it (G.L.); italo.porto@gmail.com (I.P.); 2 Università Cattolica del Sacro Cuore, 00168 Rome, Italy; francesco90@gmail.com (F.C.); josip.borovac@me.com (J.A.B.); ggmleone@yahoo.it (G.L.); valerio.destefano@unicatt.it (V.D.S.); eloisa.basile1@gmail.com (E.B.); andrea.sr.89@hotmail.it (A.S.); leonarda.galiuto@policlinicogemelli.it (L.G.); rocco.vergallo@gmail.com (R.V.); fra.canonico1984@gmail.com (F.C.); attilio94r@gmail.com (A.R.); antoniogiuseppe.rebuzzi@policlinicogemelli.it (A.G.R.); Filippo.Crea@unicatt.it (F.C.)

**Keywords:** granulocyte colony-stimulating factor, G-CSF, left ventricular remodeling, long-term effects, myocardial infarction, quality of life

## Abstract

Background: the RIGENERA trial assessed the efficacy of granulocyte-colony stimulating factor (G-CSF) in the improvement of clinical outcomes in patients with severe acute myocardial infarction. However, there is no evidence available regarding the long-term safety and efficacy of this treatment. Methods: in order to evaluate the long-term effects on the incidence of major adverse events, on the symptom burden, on the quality of life and the mean life expectancy and on the left ventricular (LV) function, we performed a clinical and echocardiographic evaluation together with an assessment using the Minnesota Living with Heart Failure Questionnaire (MLHFQ) and the Seattle Heart Failure Model (SHFM) at 10-years follow-up, in the patients cohorts enrolled in the RIGENERA trial. Results: thirty-two patients were eligible for the prospective clinical and echocardiography analyses. A significant reduction in adverse LV remodeling was observed in G-CSF group compared to controls, 9% vs. 48% (*p* = 0.030). The New York Heart Association (NYHA) functional class was lower in G-CSF group vs. controls (*p* = 0.040), with lower burden of symptoms and higher quality of life (*p* = 0.049). The mean life expectancy was significantly higher in G-CSF group compared to controls (15 ± 4 years vs. 12 ± 4 years, *p* = 0.046. No difference was found in the incidence of major adverse events. Conclusions: this longest available follow-up on G-CSF treatment in patients with severe acute myocardial infarction (AMI) showed that this treatment was safe and associated with a reduction of adverse LV remodeling and higher quality of life, in comparison with standard-of-care treatment.

## 1. Introduction

Despite advancements in pharmacological and interventional therapies have markedly reduced in-hospital morbidity and mortality, the outcome of large acute myocardial infarction (AMI) remains poor. A substantial proportion of patients suffering from a large AMI today still develop heart failure (HF), that inevitably worsens over time, impacting on quality and quantity of life. Furthermore, current therapies for post-ischemic HF do not target the primary cause of this syndrome, which is the irreversible loss of myocardial contractile tissue and cardiac fibrosis [1,2]. The use of stem cells and, in particular, of bone marrow-derived mononuclear cells (BM-MNCs), is a potentially effective strategy that could resolve this unmet need in clinical practice.

Yet, several randomized controlled trials using intramyocardial and intracoronary injections of stem cells undertaken throughout the past decade achieved mixed short-term results [3,4,5]. Furthermore, relevant meta-analyses on this topic reached no final consensus [6,7,8]. Clinical differences in patient population selection and enrollment in reported trials are identified as a potential source of bias, generating inconclusive results [9]. In addition, the stem cell approach was somewhat limited, due to the intrinsic invasive nature of the procedure, requiring bone marrow aspiration and reinfusion of these cells during a cardiac catheterization.

AMI is followed by a spontaneous mobilization of stem cells from the bone marrow and concentration of these cells in the peripheral blood positively correlated with the recovery of the left ventricular function [10]. Consequently, several studies have evaluated the potential effectiveness of a therapy with granulocyte-colony stimulating factor (G-CSF), an agent that is able to mobilize stem cells from the bone marrow, therefore, avoiding bone marrow aspiration and subsequent cell reinfusion. Nonetheless, this innovative treatment concept again provided inconclusive results [11]. In the initial report of the RIGENERA (“*Recupero dall’Infarto miocardico con G-CSF E Nuovi Esempi di Rigenerazione Avanzata”*) study, we reported a significant 5% increase in left ventricular ejection fraction (LVEF), in the absence of echocardiographic signs of ventricular dilatation in patients treated by G-CSF at 6-month follow-up, which was not observed in patients randomized to placebo [5]. However, no studies have hitherto evaluated the long-term efficacy of G-CSF treatment.

The aim of the present study was to evaluate the incidence of major adverse cardiovascular and cerebrovascular events (MACCE), the left ventricle function assessed by echocardiography, the health-related quality of life (HRQoL), as measured by the Minnesota Living with Heart Failure Questionnaire (MLHFQ), the symptom burden measured by New York Heart Association (NYHA) functional classification and the life expectancy as calculated by the Seattle Heart Failure Model (SHFM), among patients from the RIGENERA trial, at 10-year follow-up.

## 2. Materials and Methods

### 2.1. Patients and Protocol of Original RIGENERA Trial

The RIGENERA trial design and 3-month follow-up results have been previously reported [5]. In the RIGENERA study, a single-blind randomized controlled trial, 41 patients were randomized 1:2 to G-CSF therapy (*n* = 14) or optimal standard-of-care therapy (*n* = 27) from June 2003 to May 2006. The goal was to assess the potential efficacy of G-CSF administration on cardiac function in patients with a first large anterior AMI and with the LVEF <50%, despite the successful revascularization of the infarct-related artery by the percutaneous coronary intervention (PCI). The exclusion criteria were cardiogenic shock, uncontrolled myocardial ischemia or arrhythmias, malignancies, severe infections, hematologic diseases, splenomegaly on abdominal echocardiography and age >80 years. Patients randomized to G-CSF agents were treated with lenograstim (recombinant human G-CSF; Myelostim 34, Italfarmaco S.p.A., Milan, Italy), at a dose of 10 µg/kg/day for 5 days, starting ≥ 5 days after AMI and/or a complete coronary stenting. Originally, all patients should have undergone diagnostic evaluation by both conventional and myocardial contrast echocardiography (MCE) before starting therapy. However, due to the transitory warning by the European Agency for the Evaluation of Medicinal Products (EMEA) regarding the use of ultrasound agent in patients with ischemic heart disease, only 17 patients (6 in G-CSF group and 11 in control group) underwent MCE during hospitalization. The standard of care for patients assigned to G-CSF agents and control group at discharge consisted of aspirin (100 mg), clopidogrel, carvedilol, ramipril, and atorvastatin. Ticlopidine was the P2Y12 treatment used in patients that were not receiving clopidogrel. To determine the degree of stem/progenitor cell mobilization, CD34^+^ cells were assessed by flow cytometry using anti-CD34 antibody (Caltag, Burlingame, California).

### 2.2. Assessment at 10-Year Follow-Up

**Figure 1:**
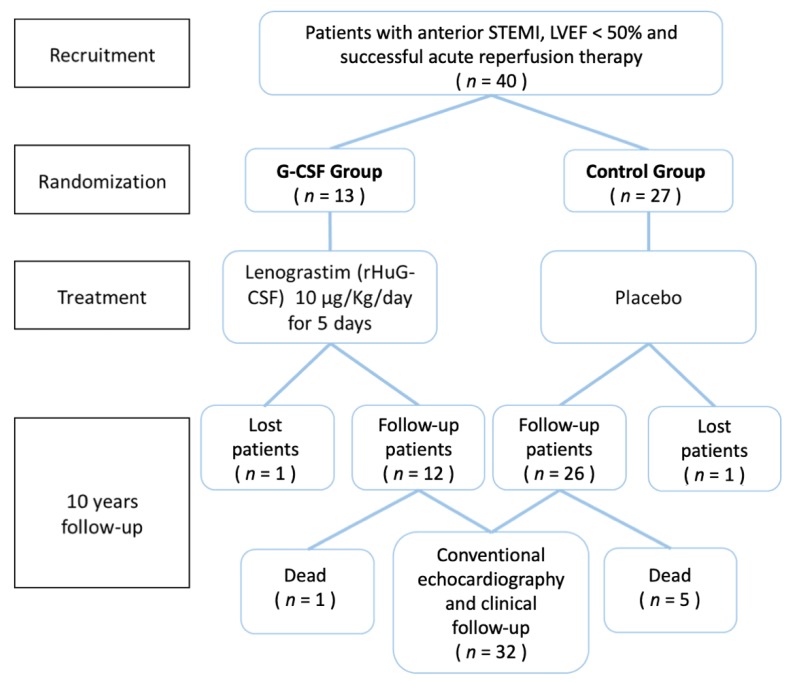
Overview of the study protocol, which shows the different phases of design, enrollment and treatment procedure of RIGENERA study and respective 10-year follow-up.

In May of 2016, the 10-year follow-up of patients from the RIGENERA trial was completed. The follow-up of these patients consisted of clinical evaluation, echocardiography, assessment of quality of life by Minnesota Living with Heart Failure Questionnaire (MLHFQ) and calculation of Seattle Heart Failure Model (SHFM) score. The study design and enrollment protocol of RIGENERA trial and 10-year follow-up is provided in the Figure 1. All patients included in the study have signed informed consent and voluntarily agreed to participate. Insertion of the data was blinded. All procedures followed were in accordance with the Declaration of Helsinki from 1975 and its revision in 2008. The Ethics Committee of the Catholic University of the Sacred Heart approved the research protocol.

#### 2.2.1. Clinical Evaluation

A clinical examination was performed for each patient, whenever possible, or patients were reached by telephone to provide information about outcomes and adverse events at each visit. The symptom burden was evaluated for each patient and classified according to the NYHA functional classification of HF.

#### 2.2.2. Echocardiography

A standard transthoracic echocardiography examination was performed in all patients to determine left ventricular ejection fraction (LVEF), left ventricular end-diastolic volume (LVEDV), left ventricular end-systolic volume (LVESV) and wall motion score index (WMSI), according to the standard guidelines of the American Society of Echocardiography (ASE) and/or European Association of Cardiovascular Imaging (EACVI). Adverse left ventricular remodeling in this study was defined as >20% increase in LVEDV from the baseline imaging and that at 10-year follow-up. The increase in LVEDV in a magnitude of at least 20% from the first postinfarction imaging is an arbitrary definition of adverse ventricular remodeling, however, it has been widely accepted as a standard in a number of follow-up studies [12,13].

#### 2.2.3. Minnesota Living with Heart Failure Questionnaire (MLHFQ)

MLHFQ scores were obtained for each patient during the 10-year follow-up. This questionnaire was specifically designed to measure the effects of heart failure (HF) and associated treatments on a patient’s HRQoL [14]. MLHFQ is a well-established and internationally validated instrument that is comprised of 21 questions rated on six-point Likert scales, determining the impact of HF on HRQoL (from 0—none to 5—very much) and encompasses two dimensions of life—physical and emotional. This questionnaire provides a total score from 0 to 105 (zero being the best and 105 marking the worst HRQoL).

#### 2.2.4. Seattle Heart Failure Model (SHFM)

The SHFM is a calculator of projected survival at baseline and after interventions for patients with HF. It was derived from a retrospective analysis of survival predictors among 1125 HF patients. A stepwise Cox-proportional hazard model was used to develop a multivariate risk model that identified age, gender, ischemic etiology of HF, NYHA functional class, LVEF, systolic blood pressure (SBP), potassium-sparing diuretic use, statin use, alopurinol use, hemoglobin concentration, % lymphocyte count, uric acid, sodium, cholesterol, and diuretic dose/kg as significant predictors of survival. This model was prospectively validated in five additional cohorts, totaling 9942 HF patients and 17307 person-years of follow-up. In conclusion, SHFM provides an accurate estimate of 1-, 2- and 5-year survival by incorporating routine laboratory, clinical, pharmacological and device parameters [15,16].

#### 2.2.5. Statistical Analysis

All data analyses were performed using Statistica 5.5 statistical package (StatSoft, Inc., Tulsa, Oklahoma). Continuous variables were compared by student *t*-test and two-way analysis of variance (ANOVA), or by their corresponding non-parametric alternatives (Mann–Whitney U test or Wilcoxon test), as appropriate. The Kolmogorov–Smirnov test determined normality of distribution. Continuous data are presented as mean ± SD. Differences with a significance (*p*) value < 0.05 were considered statistically significant.

## 3. Results

### 3.1. Clinical Outcomes

During the 10-year follow-up, three patients were lost to follow-up, making the loss to follow-up rate of 7.1%. The final population consisted of 38 patients (12 in G-CSF group and 26 in the control group). The baseline clinical, pharmacotherapy and echocardiography characteristics of a total patient sample are reported in Table 1. Medications at 10-years follow-up are reported in Table 2.

At the completion of 10-year follow-up, six deaths were recorded, resulting in the overall mortality rate of 15.8%. The cardiac mortality rate was 8.3% (one patient) in the G-CSF group and 19.2% (five patients) in the standard-of-care group, but this difference was not statistically significant (*p* = 0.41). The incidence of major adverse cardiovascular and cerebrovascular events, defined as the composite of all-cause death, recurrent myocardial infarction, in-stent restenosis, acute heart failure, urgent revascularization, and stroke did not significantly differ between the two groups at 10-year follow-up analysis. No malignant arrhythmias and no cases of cancer of any origin were diagnosed and recorded during the follow-up in both groups. Both control and G-CSF patients received the standard-of-care pharmacological treatment during the entire follow-up, without significant differences in pharmacotherapy between the two groups.

### 3.2. Echocardiographic Parameters of Ventricular Function

**Figure 2:**
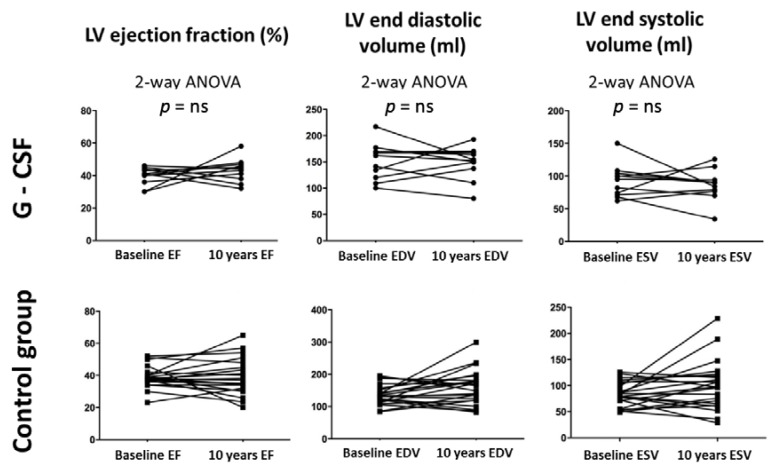
Echocardiographic parameters of patients receiving G-CSF and control group at 10-year follow-up. From left to right: left ventricular ejection fraction (LVEF), left ventricular end-diastolic volume (LVEDV) and left ventricular end-systolic volume (LVESV). Two-way ANOVA analysis was performed. NS=non-significant result.

LVEF, LVEDV, LVESV and WMSI were similar at baseline and at follow-up in both groups (Figure 2). 

**Figure 3:**
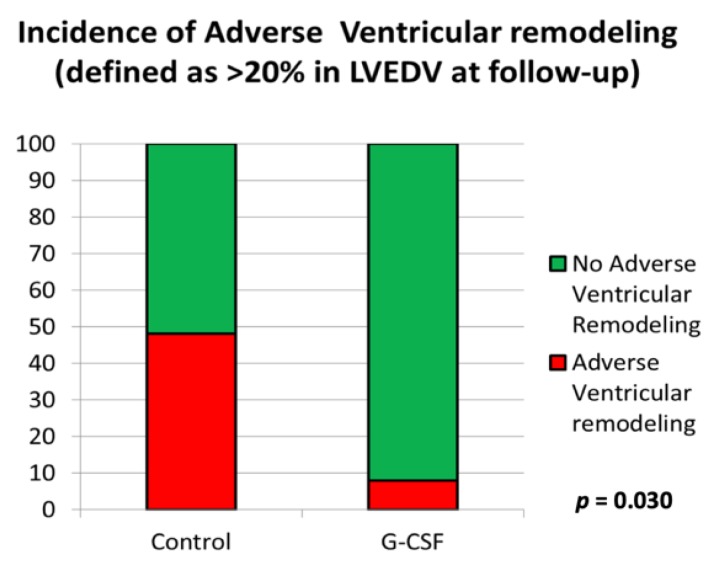
The incidence of adverse ventricular remodeling at 10-year follow-up in optimal control group versus G-CSF treatment group.

At 10-year follow-up, adverse ventricular remodeling was significantly higher in control group (10/21) than in G-CSF-treated group (1/11), 48% vs. 9%, *p* = 0.030, respectively (Figure 3). In terms of absolute numbers, there was less adverse ventricular remodeling in both groups, compared to baseline data obtained at initial enrollment in the RIGENERA study.

### 3.3. Rehospitalization Events, Life Expectancy Prognosis, Burden of Symptoms and Health-Related Quality of Life at 10-Year Follow-Up

**Figure 4:**
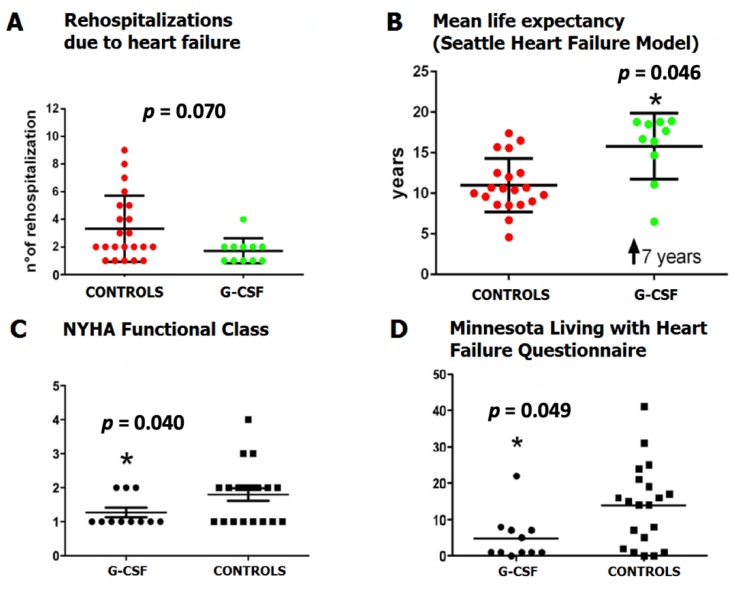
(**A**) Heart failure-related rehospitalization events, (**B**) Mean life expectancy calculated by Seattle Heart Failure Model (SHFM) between controls and G-CSF treatment group, (**C**) New York Heart Association (NYHA) functional classification of heart failure severity in G-CSF group compared to control, (**D**) MLHFQ mean score between G-CSF group and control group.

The numbers of rehospitalizations due to heart failure were higher in the control group than the G-CSF group, however, this was of marginal significance in the analysis (*p* = 0.070), with an overall incidence of 2.9% (Figure 4A). The mean life expectancy, calculated by the Seattle Heart Failure Model at 10-year follow-up, was significantly higher for G-CSF patients compared to control patients (15 ± 4 years vs. 12 ± 4 years, *p* = 0.046, respectively) (Figure 4B). 

The NYHA class at follow-up was lower in G-CSF-treated patients than in control group (*p* = 0.040) (Figure 4C). Finally, health-related quality of life assessed by the MLHF questionnaire was better in the G-CSF-treated patients than in the control group (*p* = 0.049) (Figure 4D). 

## 4. Discussion

Our data represent the longest available follow-up on the efficacy and safety of G-CSF treatment in patients with a large anterior or anterolateral ST-segment elevation myocardial infarction (STEMI). The major finding of this 10-year follow-up is that G-CSF patients, compared to patients that received optimal standard-of-care treatment, reported a significantly better quality of life and significantly lower burden of symptoms, as assessed by MLHFQ scores and NYHA functional classification of HF. This improvement was paralleled by a lower prevalence of LV remodeling and by a lower rate of readmissions for heart failure. According to the consensus definition, adverse ventricular remodeling is defined as the genomic expression, resulting in molecular, cellular, and interstitial changes that are manifested clinically as changes in size, shape, and function of the heart after cardiac injury [17]. Of note, our data show that the projected survival calculated by SHFM was significantly higher for G-CSF-treated patients, compared with the control group. This finding is obviously explained by the fact that in our study, G-CSF administration post-AMI and reperfusion had a positive impact on adverse cardiac remodeling, consequently resulting in a lower burden of symptoms and better quality of life, thus prolonging expected survival in these patients as compared to conventional treatment. 

The positive impact of G-CSF on ventricular remodeling attenuation has already been highlighted by FIRSTLINE-AMI trial and STEM-AMI trial, at 12-month and 3-year follow-up, respectively [18,19]. Indeed, the FIRSTLINE-AMI trial showed improvement in WMSI accompanied by a sustained recovery of wall thickening, LVEDD and LVEF in G-CSF-treated patients compared to the control group [18]. The three-year follow-up of patients treated with G-CSF after successful reperfusion following a large STEMI showed that no differences in mortality and MACCE were found between treatment and placebo-treated groups. Cardiac magnetic resonance imaging (CMRI) showed that infarct size did not differ between groups, however, LVEDV was significantly lower among G-CSF patients compared to those that were treated with placebo. Furthermore, a significant inverse correlation among G-CSF patients was determined between circulating CD34 cells at 30 days after reperfusion and the three-year absolute and indexed LVEDV as well as overtime [19]. These findings obtained from STEM-AMI trial showed that G-CSF treatment conferred positive effects on ventricular remodeling in a cohort of patients that suffered from a large anterior STEMI, at three-year follow-up without differences in observed clinical outcomes among groups. These results are comparable to those presented in this study. Taken together, these data suggest that a longer window of time might be necessary to ascertain the positive effects of G-CSF therapy on the damaged myocardium, and because most of the studies in this field were focused on a substantially shorter end-points, these effects might not have been detected. Finally, no study has hitherto evaluated the durability and safety of G-CSF treatment in a long-term follow-up. 

The beneficial effects of G-CSF are mediated, both by the mobilization of bone marrow-derived stem cells and by the paracrine action of the cytokine itself, in particular, the latter seems to be the main mechanism according to the emerging consensus [20]. Of note, the JAK-STAT signaling pathway has an important role in cardiac homeostasis by promoting cardiomyocyte survival, myocardial angiogenesis, infarct scar size reduction and anti-apoptotic effects [21]. The G-CSF has been described as a known activator of the JAK-STAT pathway and by this action, it prevented cardiac remodeling after AMI by mobilizing bone marrow stem cells. Furthermore, G-CSF induced the expression of anti-apoptotic proteins and inhibited apoptosis in cardiomyocytes affected by infarction [22]. On the opposite end, a treatment with STAT3 inhibitor completely abolished the beneficial effects of G-CSF on cardiac function, remodeling, cardiomyocyte degeneration and fibrosis [22,23].

The original RIGENERA study demonstrated that patients treated with G-CSF had higher EF and lower LVEDV at 5-month follow-up, as compared to controls in the absence of significant differences in clinical end-points [5]. This 10-year follow-up study seems to show that the functional differences initially observed between the two groups might translate into a better clinical outcome. Moreover, this data suggest that a degree of adverse ventricular remodeling might be a more important prognostic predictor than ejection fraction in this population and the one that reflects the clinical improvements and projected survival that were observed in follow-up analysis. This concept was emphasized by Pfeffer and Braunwald, that concluded how attenuation of ventricular enlargement and degree of ventricular remodeling after AMI directly correlated with survival prolongation [24]. 

## 5. Conclusions

This study shows that in patients with large STEMI and LV dysfunction, treatment with G-CSF during the index admission is safe and is associated with beneficial effects on LV remodeling, symptom burden, life expectancy and quality of life at 10-year follow-up. Future large randomized studies powered for long-term analyses are required to confirm the effects of G CSF in the context of myocardial repair after large AMI. In this regard, there are two major Phase III trials, the STEM-AMI trial (https://clinicaltrials.gov/ct2/show/NCT01969890) and RIGENERA 2.0 trial (https://clinicaltrials.gov/ct2/show/NCT02502747), designed by our group to answer these challenging questions [19,25]. The RIGENERA 2.0 trial, ongoing and conducted by our group, will recruit 120 patients with STEMI and LVEF ≤45%, randomized 1:1 to receive either subcutaneous recombinant human G-CSF and intravenous infusion of sulfur hexafluoride (SF6) to improve stem cell homing or placebo [25].

## 6. Limitations

Due to the small number of patients analyzed at the 10-year follow-up, the results of the present study should be interpreted with caution. No power calculation could be performed because of a lack of previous studies with this long-term follow-up. Thus, the final sample size is the result of the original design of the trial, previously published elsewhere [5]. 

G-CSF patients and controls patients were properly matched for age, sex, medication and risk factors at baseline and at the 10-year follow-up period: none of these variables were independently associated with LV remodeling on the univariate analysis (data not shown). However, it should acknowledge that we are not able to provide any information regarding the up-titration/optimization of therapy which possibly occurred during the time period analyzed. These limitations imply two dominant methodological issues that cannot be eluded. First, several variables, together with the G-CSF treatment, might concur with the outcomes observed. Second, it is impossible in this type of study to determine a cause–effect relationship.

## Figures and Tables

**Table 1:** Baseline clinical, medications and echocardiography characteristics of patients randomized to control standard-of-care vs patients randomized to granulocyte-colony stimulating factor (G-CSF) treatment at 10-year follow-up.

Variables	Control Group *n* = 26	G-CSF Group *n* = 12	*p*
**Clinical Characteristics**			
Age (years ± SD)	66 ± 11	63 ± 11	0.35
Male (*n*, (%))	21 (81)	12 (100)	0.10
Female (*n*, (%))	5 (19)	0 (0)	0.10
Hypertension (*n*, (%))	18 (69)	6 (50)	0.25
Current smoker (*n*, (%))	17 (65)	9 (75)	0.55
Dyslipidemia (*n*, (%))	10 (39)	8 (67)	0.11
Diabetes mellitus (*n*, (%))	4 (15)	3 (25)	0.48
Family history of CAD (*n*, (%))	7 (27)	7 (59)	0.06
STEMI (*n*, (%))	26 (100)	12 (100)	1
Primary percutaneous intervention (*n*, (%))	26 (100)	12 (100)	1
Thrombolysis (*n*, (%))	11 (42)	7 (58)	0.36
Troponin T (mean ± SD) (ng/mL)	14.7 ± 8.55	11.3 ± 7.1	0.20
Multivessel CAD (*n*, (%))	2 (8)	0 (0)	0.32
**Pharmacotherapy**			
Beta-blockers (*n*, (%))	25 (96)	11 (92)	0.56
ACE inhibitors (*n*, (%))	22 (85)	12 (100)	0.15
Statins (*n*, (%))	22 (85)	11 (92)	0.55
Aspirin (*n*, (%))	25 (96)	12 (100)	0.49
ARBs (*n*, (%))	5 (19)	3 (25)	0.69
Diuretics (*n*, (%))	13 (50)	8 (67)	0.34
Heparin (*n*, (%))	1 (4)	1 (8)	0.56
Clopidogrel (*n*, (%))	14 (54)	10 (83)	0.08
**Echocardiography at enrollment**			
LV Ejection Fraction (LVEF) (%)	39.1 ± 8	39.3 ± 5	0.95
LV End Diastolic Volume (LVEDV) (mL)	141 ± 35	149 ± 34	
LV End Systolic Volume (LVESV) (mL)	86 ± 24	91 ± 24	
Wall Motion Score Index (WMSI)	2.1 ± 0.4	2.0 ± 0.2	

**Abbreviations:** ACE-angiotensin-converting-enzyme, CAD-coronary artery disease, ARBs-angiotensin II receptor blockers, STEMI-ST-segment Elevation Myocardial Infarction.

**Table 2:** Medications at 10-years follow-up.

	Control Group *n* = 21	G-CSF Group *n* = 11	*p*
**Medications at 10-years follow-up**			
Beta-blockers (*n*, (%))	19 (90)	9 (82)	0.48
ACE inhibitors/ARBs (*n*, (%))	18 (86)	10 (91)	0.67
Statins (*n*, (%))	18 (86)	9 (82)	0.77
Aspirin (*n*, (%))	20 (95)	10 (91)	0.63
Diuretics (*n*, (%))	14 (67)	7 (64)	0.86
P_2_Y_12_ inhibitor (*n*, (%))	3 (14)	2 (18)	0.77

**Abbreviations:** ACE-angiotensin-converting-enzyme, ARBs-angiotensin II receptor blockers.

## Fundings

This work was supported by Banca d’Italia (unrestricted grant) and by Ministero dell’Istruzione, dell’Università e della Ricerca (MIUR- PRIN 2010).
